# Supplementary material for: Residue 6.43 defines receptor function in class F GPCRs
Source: Nat Commun. 2021 Jun 24;12:3919. doi: 10.1038/s41467-021-24004-z (PMC8225760; doi:10.1038/s41467-021-24004-z)
Supplement: Supplementary file 1 — Supplementary Information [file 41467_2021_24004_MOESM1_ESM.pdf]

**Supplementary Figures 1-12, Supplementary Table 1**

# Residue 6.43 defines receptor function in Class F GPCRs

**Authors:** Ainoleena Turku[1,2], Hannes Schihada[1,#], Pawel Kozielewicz[1,#], Carl-Fredrik Bowin[1], Gunnar Schulte[1]*

**Supplementary Figure 1**. Sequence logos for transmembrane region 6 (TM6) of Class F receptors. TM6 was extracted from a large-scale alignment of Class F receptors using the homology model of $FZD_6$ as guide.[1] Residues are numbered using the $FZD_6$ Ballesteros-Weinstein residue identifiers. Residue $P/F^{6.43}$ is marked with a red arrow.

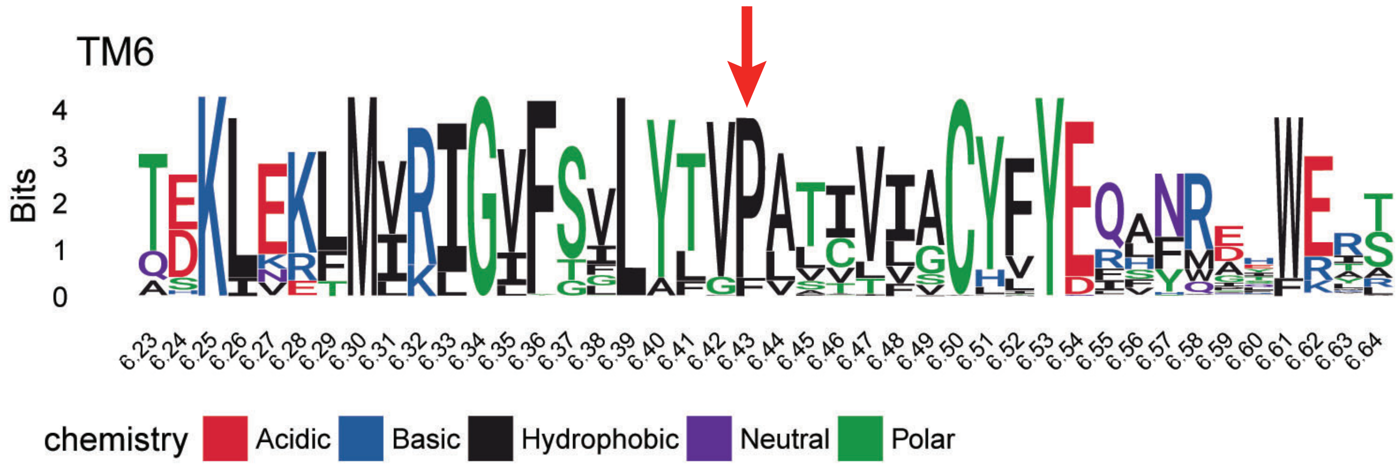
TM6
Bits
0
1
2
3
4
6.23 6.24 6.25 6.26 6.27 6.28 6.29 6.30 6.31 6.32 6.33 6.34 6.35 6.36 6.37 6.38 6.39 6.40 6.41 6.42 6.43 6.44 6.45 6.46 6.47 6.48 6.49 6.50 6.51 6.52 6.53 6.54 6.55 6.56 6.57 6.58 6.59 6.60 6.61 6.62 6.63 6.64
chemistry
Acidic
Basic
Hydrophobic
Neutral
Polar

**Supplementary Figure 2**. Protein backbone RMSDs for $FZD_6$ wild type, $FZD_6$ $P^{6.43}F$, SMO wild type and SMO $F^{6.43}P$ simulations. Thick traces indicate the moving average smoothed over a 1 ns window and thin traces represent raw data. RMSD values are calculated referred to the last frame of the 50 ns equilibration run (defined as $t = 0$). Replica 1 is shown in blue, replica 2 in pink, replica 3 in raspberry, and replica 4 in violet.

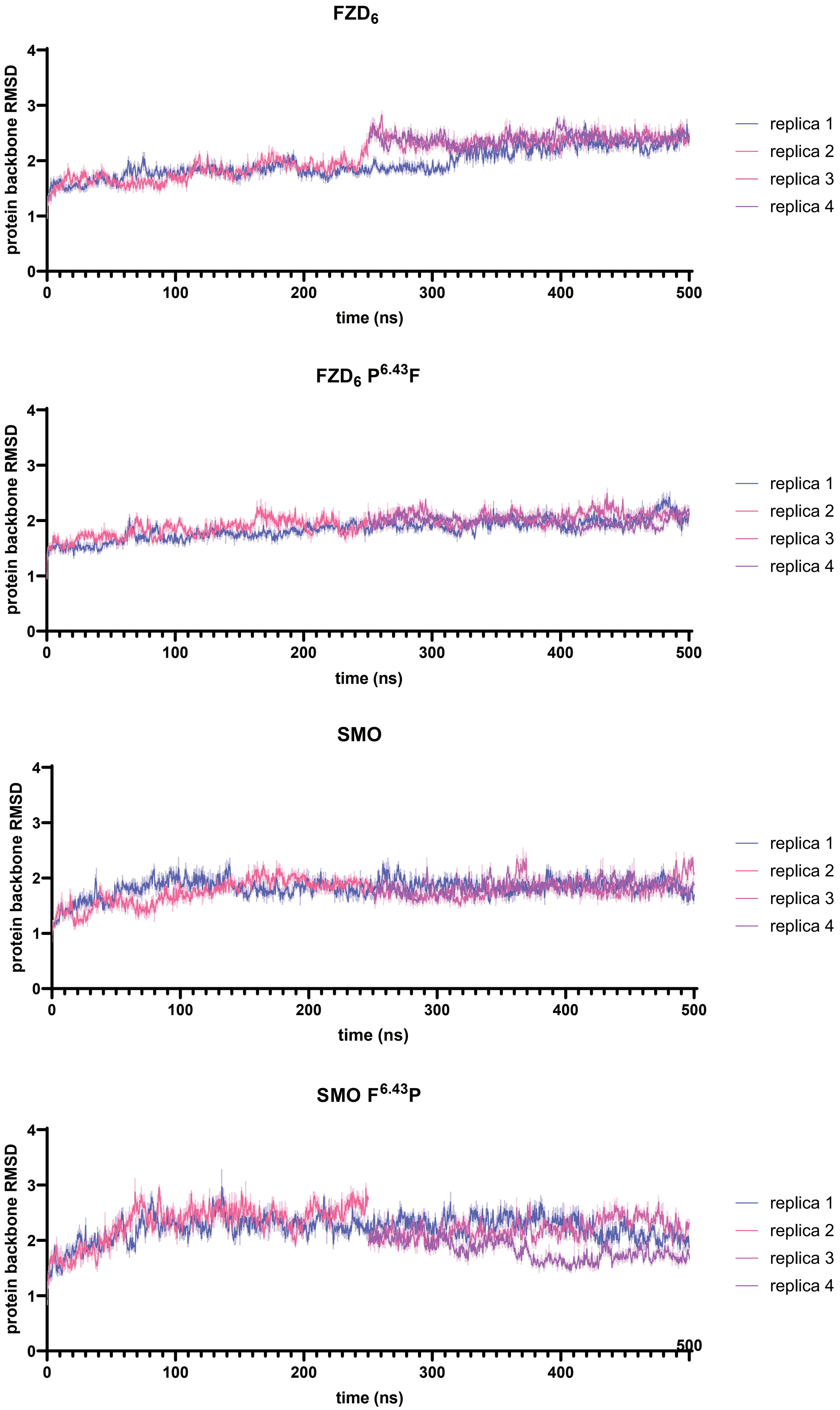
FZD6
protein backbone RMSD
time (ns)
replica 1
replica 2
replica 3
replica 4
FZD6 P6.43F
protein backbone RMSD
time (ns)
replica 1
replica 2
replica 3
replica 4
SMO
protein backbone RMSD
time (ns)
replica 1
replica 2
replica 3
replica 4
SMO F6.43P
protein backbone RMSD
time (ns)
replica 1
replica 2
replica 3
replica 4

**Supplementary Figure 3**. Proline puckering. The $\chi_2$ torsion angles of the proline residues throughout the simulation trajectories of $FZD_6$ wild type and SMO $F^{6.43}P$ to monitor the ring puckering states of the $P^{6.43}$ (as described in [2]). Replica 1 is shown in blue, replica 2 in raspberry, replica 3 in pink, and replica 4 in violet. In the $FZD_6$ simulations, $P^{6.43}$ is in an up-puckering state in approximately 85% of the simulation frames, which is in line with the puckering states observed in the trans-prolines located in the middle of alpha helices in PDB.[3] The up-puckering state allows more relaxed (i.e., less kinked) alpha helix conformation than the down-puckering state, and thus our $FZD_6$ simulations are not likely to over-estimate the kink. In SMO $F^{6.43}P$ simulations, both proline puckering conformations are approximately equally present (51% in up and 49% in down conformation), as in those trans-prolines in PDB that are not located in alpha helices.[3] This might be due to the presence of the glycine residue at position 6.42 of SMO, which, due to lack of a side chain, provides more space for the neighboring $P^{6.43}$ to access the down-puckering state. Despite this additional flexibility, the SMO $F^{6.43}P$ simulations are sampling both puckering states equally.

**$FZD_6$: Proline puckering**

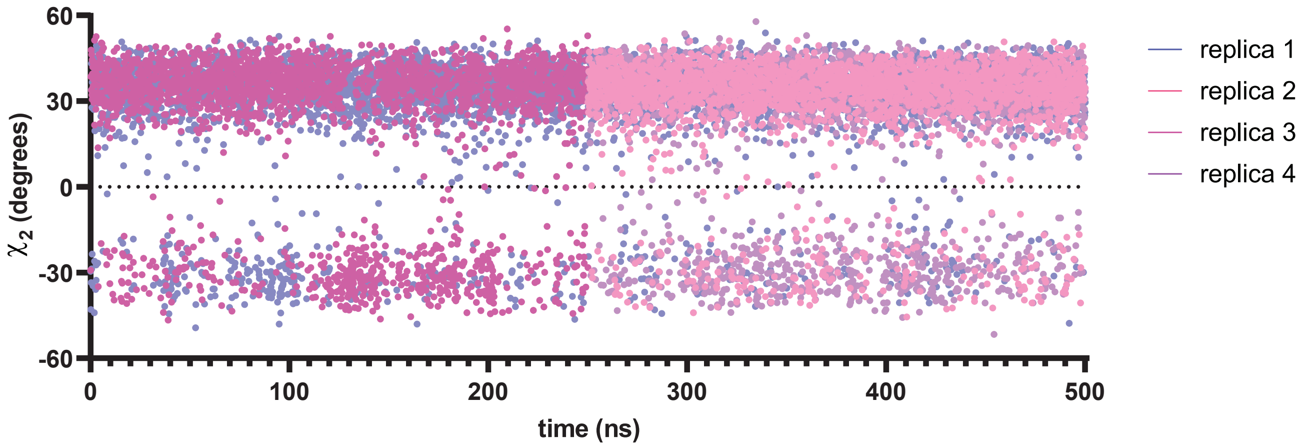


**SMO $F^{6.43}P$: Proline puckering**

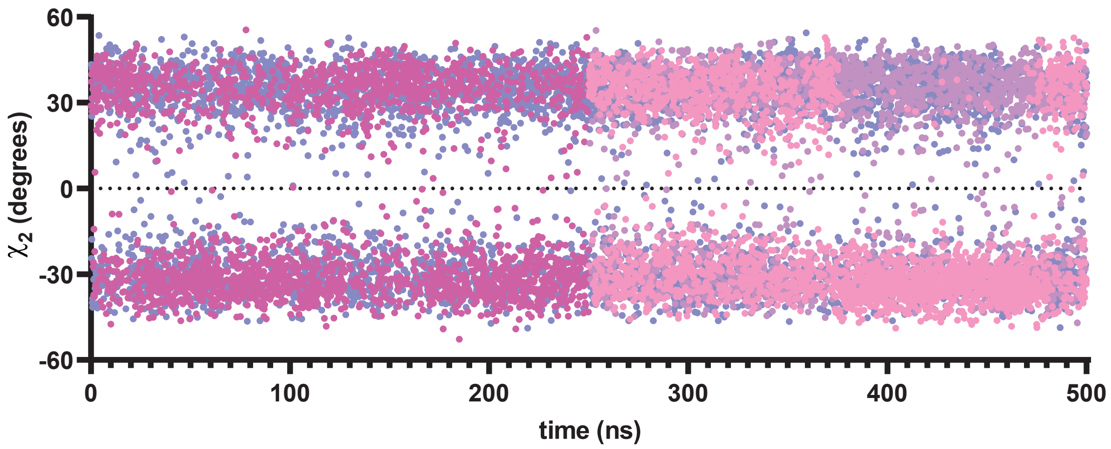

**Supplementary Figure 4**. $FZD_6$-mini$G_i$ simulations. An overview of $FZD_6$-SAG1.3-mini$G_i$ complex at $t_0$ (i.e. after 50 ns of equilibriation MD; upper left panel). The receptor is shown as light orange cartoon, mini$G_i$ as white cartoon and SAG1.3 as orange sticks. Carbon atoms are shown in orange, nitrogen in blue, oxygen in red, sulphur in yellow, and chlorine in green. Superimposition of the original $FZD_6$ wild type conformation (the last frame of the 500 ns simulation; white) and the receptors of the last frames of the three $FZD_6$-mini$G_i$ simulation replicas (light orange; upper right panel). The TM6 kink seen in all these receptor conformations and the TM2 bulge present only at the original $FZD_6$ conformation are marked with pink arrows. Distances between the counterparts of the aromatic network monitored from the $FZD_6$-mini$G_i$ trajectories (middle panels). Replica 1 is shown in blue, replica 2 in pink, and replica 3 in raspberry. Thick traces indicate the moving average smoothed over a 1 ns window and thin traces the raw data. The *x*-axes are relative to those presented in Supplementary Fig. 8 and 9. 7TM pocket volumes of the $FZD_6$-mini$G_i$ trajectories (orange) compared with $FZD_6$ wild type (white) and $FZD_6$ $P^{6.43}F$ trajectories (grey) as in Fig. 2d (lower left panel). The $\chi_2$ torsion angles of the proline residues throughout the simulation trajectories of $FZD_6$-mini$G_i$ (lower right panel). Replica 1 is shown in blue, replica 2 in pink, and replica 3 in raspberry. The puckering state distribution follows that of the $FZD_6$ wild type (without mini$G_i$) presented in Supplementary Fig. 3.

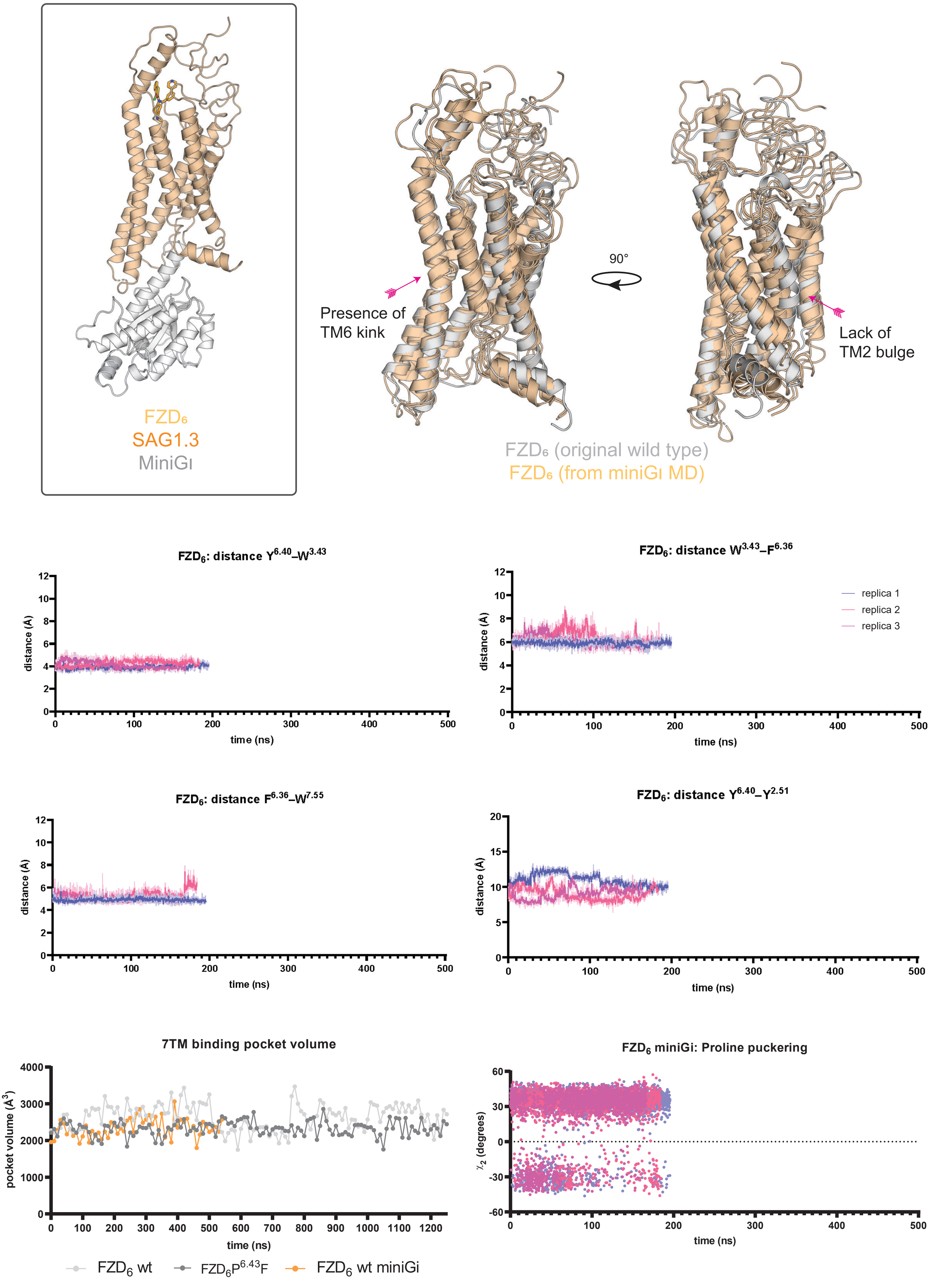
FZD6
SAG1.3
MiniGi
Presence of TM6 kink
90°
Lack of TM2 bulge
FZD6 (original wild type)
FZD6 (from miniGi MD)
FZD6: distance Y6.40–W3.43
FZD6: distance W3.43–F6.36
replica 1
replica 2
replica 3
FZD6: distance F6.36–W7.55
FZD6: distance Y6.40–Y2.51
distance (Å)
time (ns)
7TM binding pocket volume
pocket volume (Å3)
FZD6 wt
FZD6P6.43F
FZD6 wt miniGi
FZD6 miniGi: Proline puckering
χ2 (degrees)

**Supplementary Figure 5**. Schematic representation of the assay principle (left). BODIPY-cyclopamine binding to wild type (black) and $F^{6.43}P$ (red) ΔCRD HiBIT-SMO (right). BODIPY-cyclopamine binding to ΔCRD SMO was assessed by the NanoBiT/BRET binding assay. Saturation curves are presented as sigmoidal curves with logarithmic BODIPY-cyclopamine concentrations. Graphs present net NanoBRET values. Data points are presented as mean ± SEM from n=4 individual experiments performed at least in duplicates (10 pM and 300 nM concentrations were used in two individual experiments – please refer to the Source Data file). Curves were fit according to a three-parameter model. Source data are provided as a Source Data file.

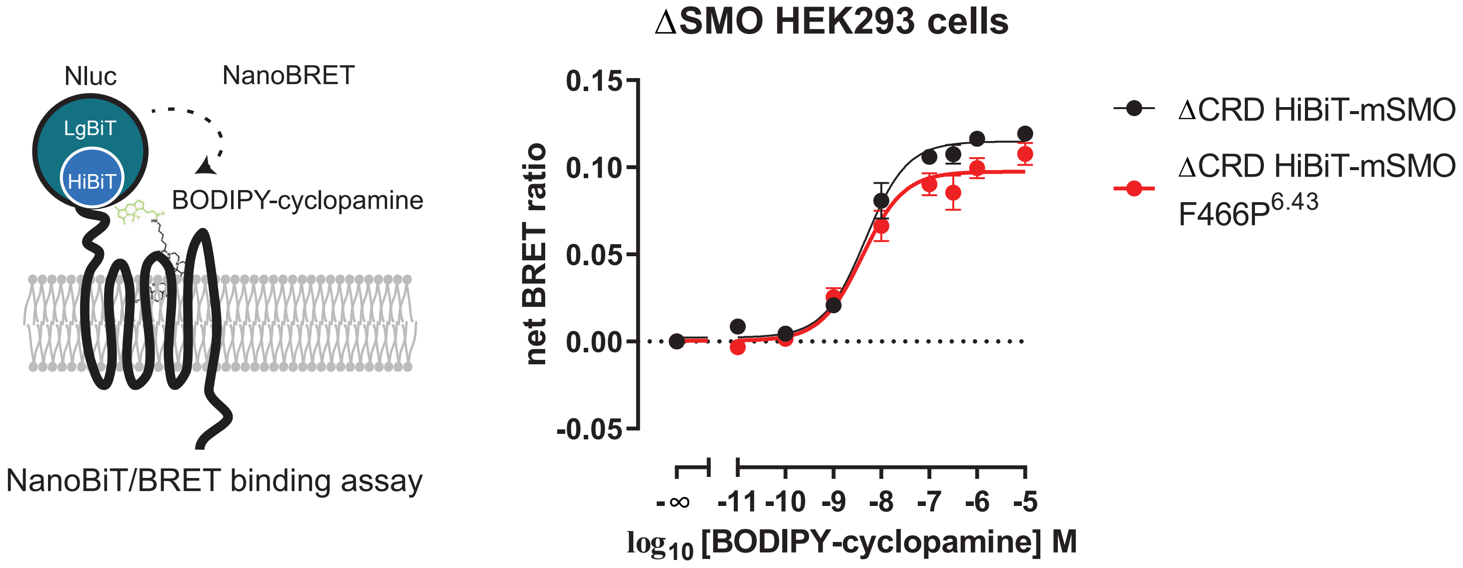
Nluc
NanoBRET
LgBiT
HiBiT
BODIPY-cyclopamine
NanoBiT/BRET binding assay
ΔSMO HEK293 cells
net BRET ratio
0.15
0.10
0.05
0.00
-0.05
-∞
-11 -10 -9 -8 -7 -6 -5
log10 [BODIPY-cyclopamine] M
ΔCRD HiBiT-mSMO
ΔCRD HiBiT-mSMO F466P6.43

**Supplementary Figure 6**. **a)** Schematic representation of the NanoBit surface expression assay. **b)** Surface expression of the HiBiT-SMO, **c)** HiBiT-$FZD_6$ and **d)** ΔCRD HiBiT-SMO constructs. Data points are presented as mean ± SEM from n=4 individual experiments for ΔCRD HiBiT-SMO wild type, ΔCRD HiBiT-SMO $F^{6.43}P$ and HiBiT-$FZD_6$ $P^{6.43}F$, and n=5 individual experiments for the other HiBiT receptor constructs. **e)** Surface expression of SNAP-tagged receptor constructs. The cell surface expression of the constructs was assessed by SNAP-surface Alexa Fluor 647 staining. The staining of pcDNA-transfected cells was used as a baseline. Subsequently, for each receptor the values were normalized to the wild type. Data are presented as mean ± SEM of 4 independent experiments. $P = 0.0064$ for $FZD_6$ and $P = 0.0255$ for SMO. **f)** Surface expression of receptor-Nluc constructs. Data are presented as mean ± SEM of 3 independent experiments. Data were analysed for each receptor wild type/mutant using paired two-tailed t-test. $*P < 0.05$, $**P < 0.01$, ns = not significant. Source data are provided as a Source Data file.

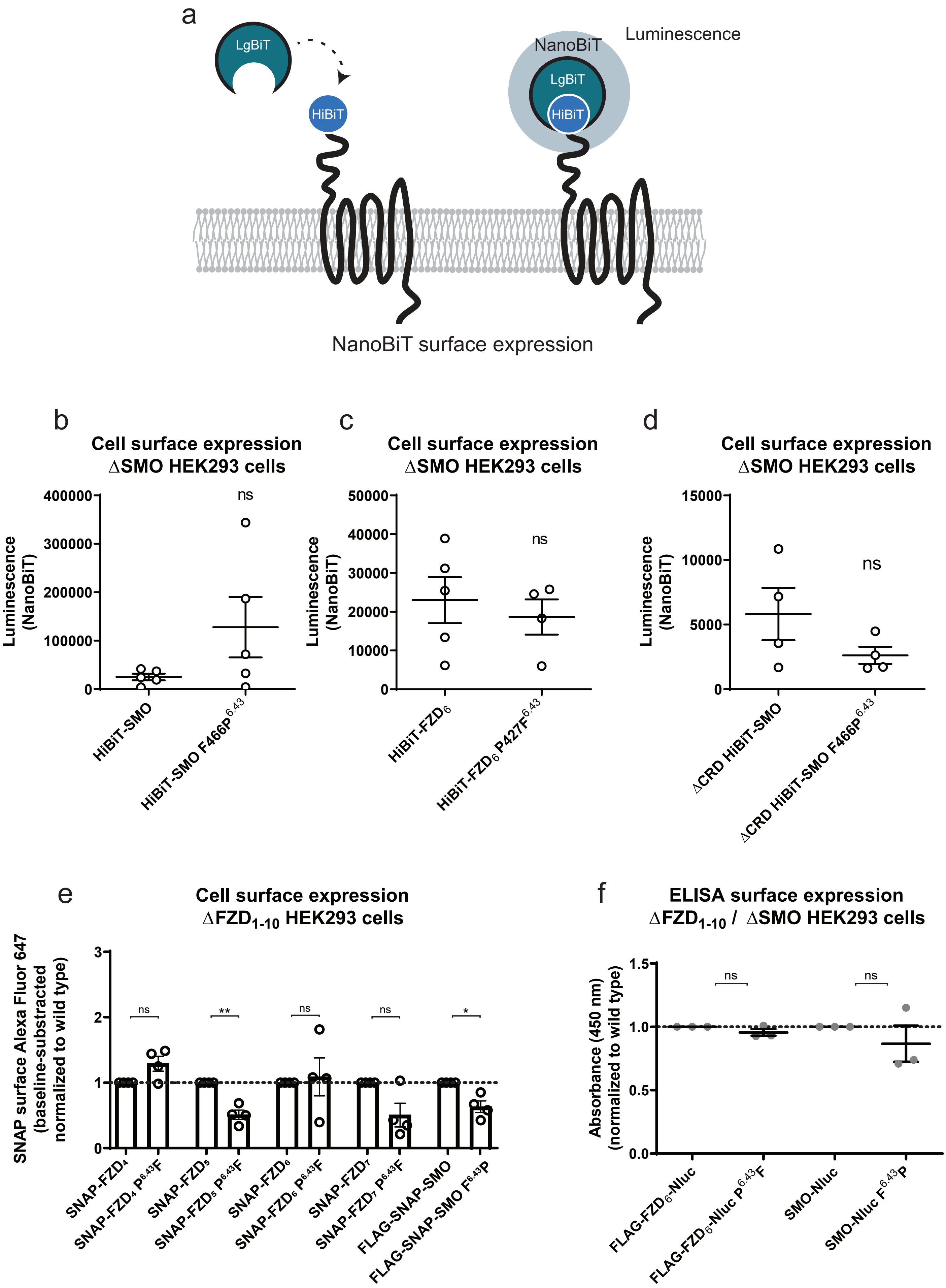
a
LgBiT
HiBiT
NanoBiT
LgBiT
HiBiT
Luminescence
NanoBiT surface expression
b
Cell surface expression
ΔSMO HEK293 cells
Luminescence (NanoBiT)
400000
300000
200000
100000
0
ns
HiBiT-SMO
HiBiT-SMO F466P$^{6.43}$
c
Cell surface expression
ΔSMO HEK293 cells
Luminescence (NanoBiT)
50000
40000
30000
20000
10000
0
ns
HiBiT-FZD$_6$
HiBiT-FZD$_6$ P427F$^{6.43}$
d
Cell surface expression
ΔSMO HEK293 cells
Luminescence (NanoBiT)
15000
10000
5000
0
ns
ΔCRD HiBiT-SMO
ΔCRD HiBiT-SMO F466P$^{6.43}$
e
Cell surface expression
ΔFZD$_{1-10}$ HEK293 cells
SNAP surface Alexa Fluor 647 (baseline-substracted normalized to wild type)
3
2
1
0
ns
**
ns
ns
*
SNAP-FZD$_4$
SNAP-FZD$_4$ P$^{6.43}$F
SNAP-FZD$_5$
SNAP-FZD$_5$ P$^{6.43}$F
SNAP-FZD$_6$
SNAP-FZD$_6$ P$^{6.43}$F
SNAP-FZD$_7$
SNAP-FZD$_7$ P$^{6.43}$F
FLAG-SNAP-SMO
FLAG-SNAP-SMO F$^{6.43}$P
f
ELISA surface expression
ΔFZD$_{1-10}$ / ΔSMO HEK293 cells
Absorbance (450 nm) (normalized to wild type)
1.5
1.0
0.5
0.0
ns
ns
FLAG-FZD$_6$-Nluc
FLAG-FZD$_6$-Nluc P$^{6.43}$F
SMO-Nluc
SMO-Nluc F$^{6.43}$P

**Supplementary Figure 7**. Extension of the molecular switch in crystal structures of the class F receptors. Sequence alignment of the extended aromatic network in human class F receptors. The discussed amino acid residues are marked with pink arrows and Ballesteros-Weinstein numbering and Pro/Phe$^{6.43}$ with pink outline (upper left panel). Crystal/CryoEM structures of $FZD_4$ and $FZD_5$ (upper right panel), homology models of active-like and inactive $FZD_6$ (middle panels) and crystal/CryoEM structures of the active and inactive SMO (lower panels). Receptors are shown as cartoon and the discussed amino acid residues as sticks. Color code is as follows: pink cartoon: inactive $FZD_4$, white cartoon: active $FZD_6$, light pink cartoon: inactive $FZD_6$, light violet cartoon: active SMO, dark violet cartoon: inactive SMO, red sticks: oxygen, dark blue sticks: nitrogen, other sticks: carbon. Green dashes mark possible hydrogen bonds.

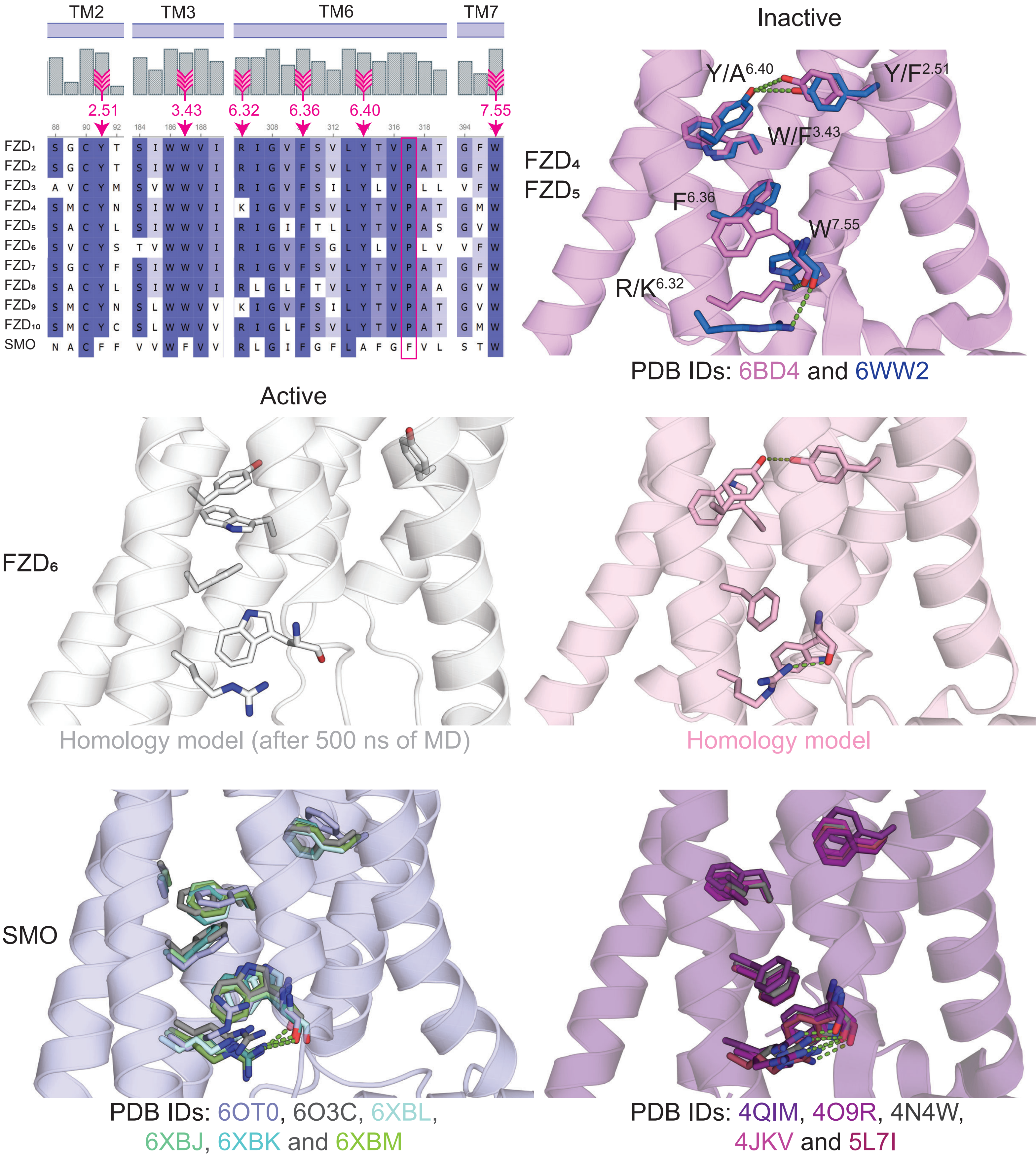

TM2
TM3
TM6
TM7
2.51
3.43
6.32
6.36
6.40
7.55
88 90 92
184 186 188
308 312 316 318
394
FZD1 S G C Y T S I W W V I R I G V F S V L Y T V P A T G F W
FZD2 S G C Y T S I W W V I R I G V F S V L Y T V P A T G F W
FZD3 A V C Y M S V W W V I R I G V F S I L Y L V P L L V F W
FZD4 S M C Y N S I W W V I K I G V F S V L Y T V P A T G M W
FZD5 S A C Y L S I W W V I R I G I F T L L Y T V P A S G V W
FZD6 S V C Y S T V W W V I R I G V F S G L Y L V P L V V F W
FZD7 S G C Y F S I W W V I R I G V F S V L Y T V P A T G F W
FZD8 S A C Y L S I W W V I R L G L F T V L Y T V P A A G V W
FZD9 S M C Y N S L W W V V K I G V F S I L Y T V P A T G V W
FZD10 S M C Y C S L W W V V R I G L F S V L Y T V P A T G M W
SMO N A C F F V V W F V V R L G I F G F L A F G F V L S T W
Inactive
Y/A6.40
Y/F2.51
W/F3.43
FZD4
FZD5
F6.36
W7.55
R/K6.32
PDB IDs: 6BD4 and 6WW2
Active
FZD6
Homology model (after 500 ns of MD)
Homology model
SMO
PDB IDs: 6OT0, 6O3C, 6XBL, 6XBJ, 6XBK and 6XBM
PDB IDs: 4QIM, 4O9R, 4N4W, 4JKV and 5L7I

**Supplementary Figure 8**. Distances between the counterparts of the aromatic network of $FZD_6$ wild type and $FZD_6$ $P^{6.43}F$ (left panels) and SMO wild type and SMO $F^{6.43}P$ (right panels) throughout the MD frames. Replica 1 is shown in blue, replica 2 in pink, replica 3 in raspberry, and replica 4 in violet. Thick traces indicate the moving average smoothed over a 1 ns window and thin traces the raw data.

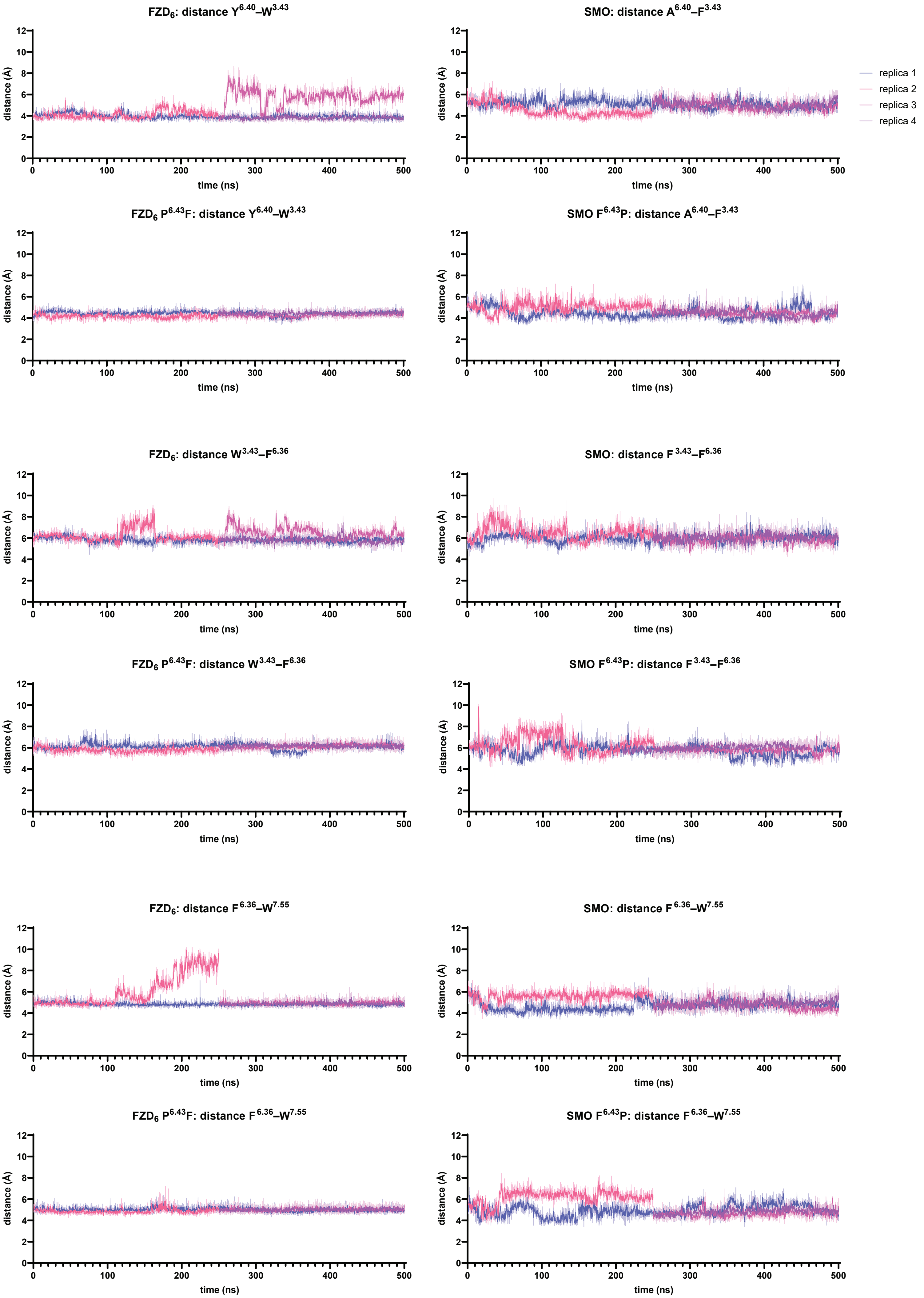

$FZD_6$: distance $Y^{6.40}$–$W^{3.43}$
SMO: distance $A^{6.40}$–$F^{3.43}$
$FZD_6$ $P^{6.43}F$: distance $Y^{6.40}$–$W^{3.43}$
SMO $F^{6.43}P$: distance $A^{6.40}$–$F^{3.43}$
$FZD_6$: distance $W^{3.43}$–$F^{6.36}$
SMO: distance $F^{3.43}$–$F^{6.36}$
$FZD_6$ $P^{6.43}F$: distance $W^{3.43}$–$F^{6.36}$
SMO $F^{6.43}P$: distance $F^{3.43}$–$F^{6.36}$
$FZD_6$: distance $F^{6.36}$–$W^{7.55}$
SMO: distance $F^{6.36}$–$W^{7.55}$
$FZD_6$ $P^{6.43}F$: distance $F^{6.36}$–$W^{7.55}$
SMO $F^{6.43}P$: distance $F^{6.36}$–$W^{7.55}$
distance (Å)
time (ns)
0
2
4
6
8
10
12
100
200
300
400
500
replica 1
replica 2
replica 3
replica 4

**Supplementary Figure 9**. Distances between Tyr$^{6.40}$ and Tyr$^{2.51}$ in $FZD_6$ (upper panel) and $FZD_6$ P$^{6.43}$F (lower panel) throughout the MD frames. Replica 1 is shown in blue, replica 2 in pink, replica 3 in raspberry, replica 4 in violet and ligand-free simulation in black. Thick traces indicate the moving average smoothed over a 1 ns window and thin traces the raw data.

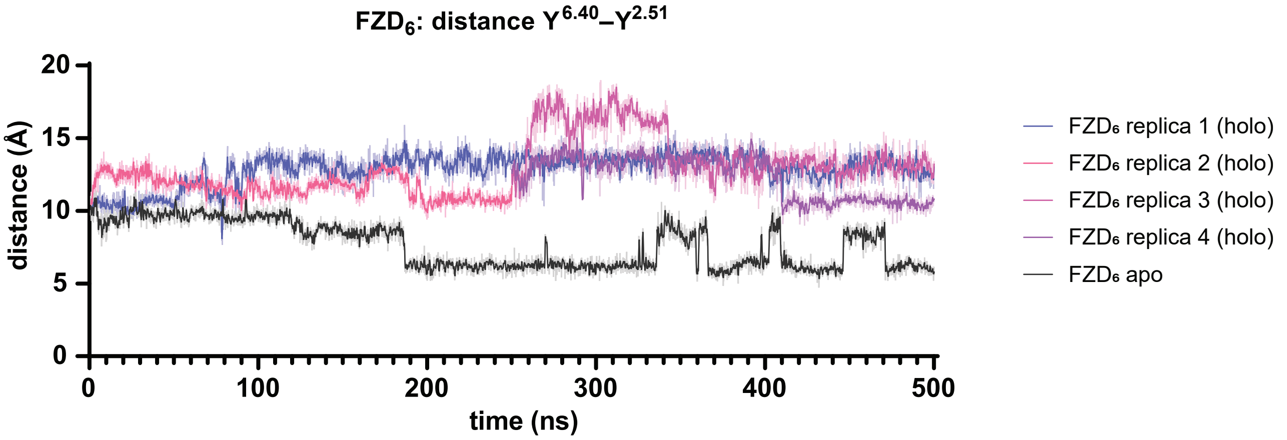
FZD6: distance Y6.40–Y2.51
distance (Å)
time (ns)
0
5
10
15
20
0
100
200
300
400
500
FZD6 replica 1 (holo)
FZD6 replica 2 (holo)
FZD6 replica 3 (holo)
FZD6 replica 4 (holo)
FZD6 apo

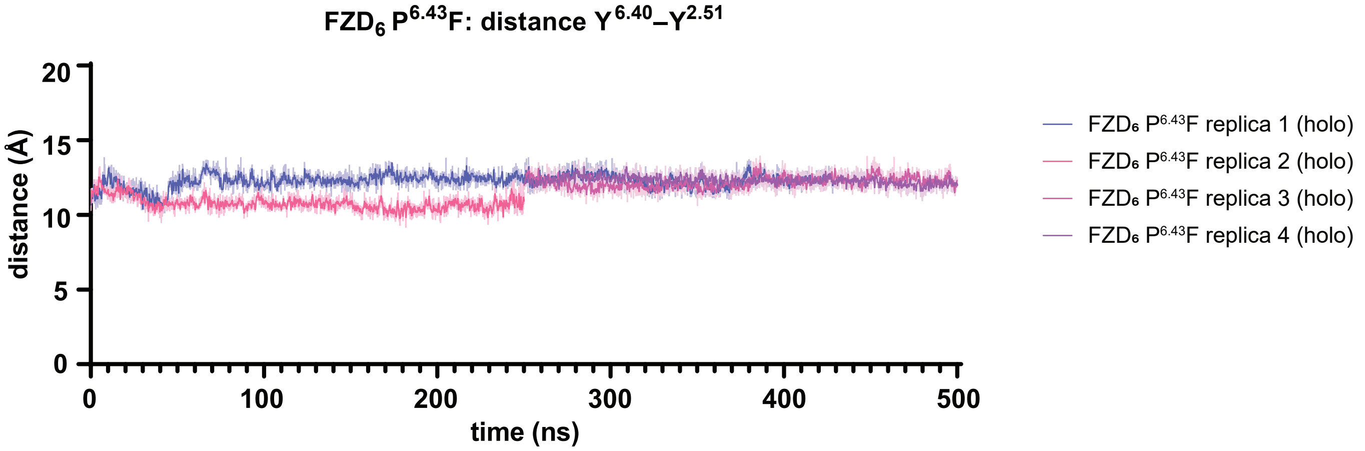
FZD6 P6.43F: distance Y6.40–Y2.51
distance (Å)
time (ns)
0
5
10
15
20
0
100
200
300
400
500
FZD6 P6.43F replica 1 (holo)
FZD6 P6.43F replica 2 (holo)
FZD6 P6.43F replica 3 (holo)
FZD6 P6.43F replica 4 (holo)

**Supplementary Figure 10.** Schematic presentation of the mGsi assay (upper panel). mGsi recruitment to SMO wild type and $F^{6.43}P$ mutant (light and dark violet, respectively; lower left panel) and $FZD_6$ wild type and $P^{6.43}F$ mutant (light and dark grey, respectively; lower right panel) in ΔSMO HEK293A cells with transiently transfected C-terminally Nluc-tagged receptors. Data are represented as mean ± SEM of n=4 independent experiments for SMO wild type and $F^{6.43}P$, n=5 independent experiments for $FZD_6$ wild type and n=10 independent experiments for $FZD_6$ $P^{6.43}F$ constructs and fitted to a bell-shaped model. See Supplementary Figure 6f for the cell surface expression data of the receptor-Nluc constructs. Source data are provided as a Source Data file.

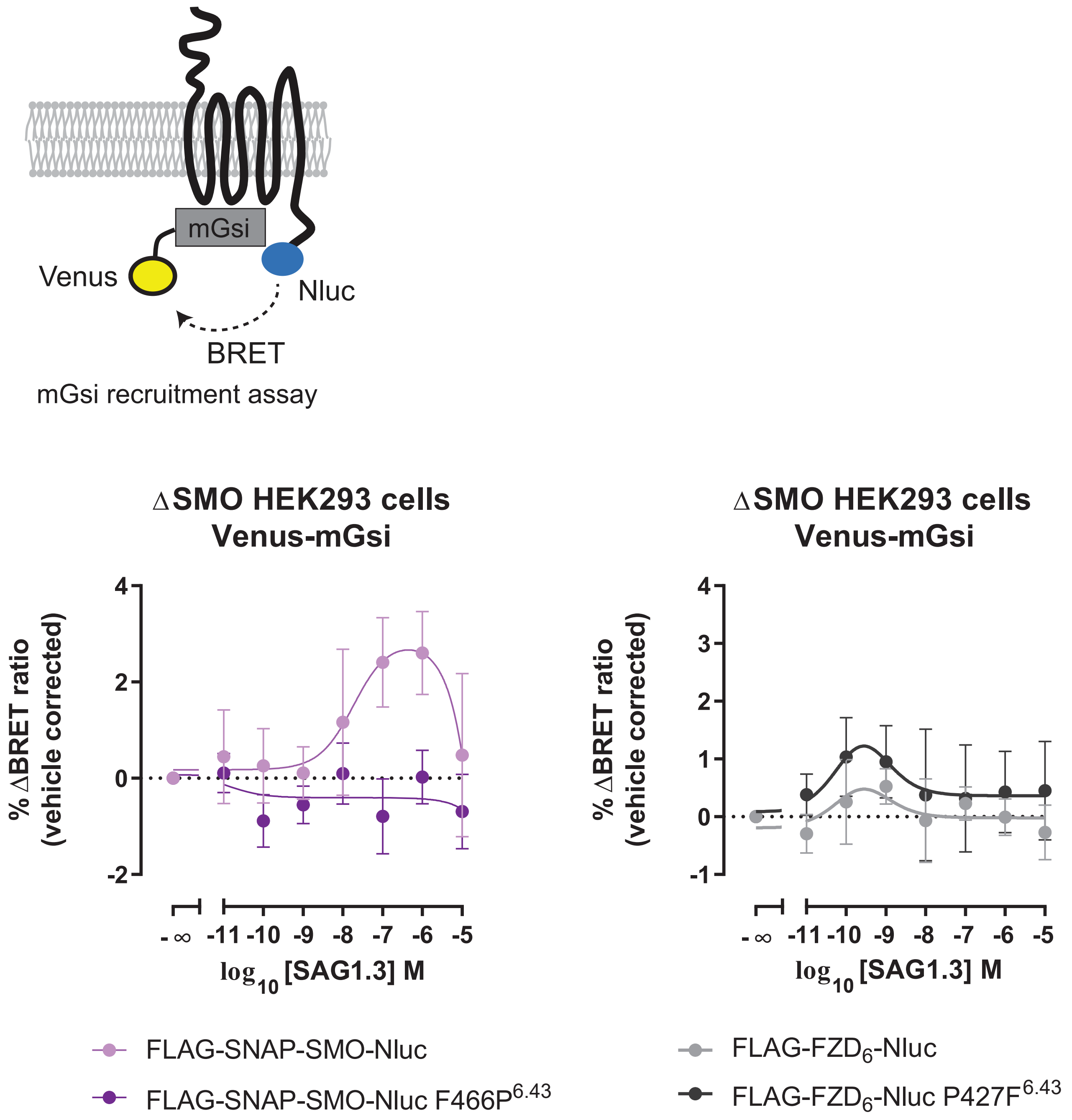
mGsi
Venus
Nluc
BRET
mGsi recruitment assay
∆SMO HEK293 cells
Venus-mGsi
% ∆BRET ratio
(vehicle corrected)
4
2
0
-2
- ∞
-11 -10 -9 -8 -7 -6 -5
log10 [SAG1.3] M
∆SMO HEK293 cells
Venus-mGsi
% ∆BRET ratio
(vehicle corrected)
4
3
2
1
0
-1
- ∞
-11 -10 -9 -8 -7 -6 -5
log10 [SAG1.3] M
FLAG-SNAP-SMO-Nluc
FLAG-SNAP-SMO-Nluc F466P6.43
FLAG-FZD6-Nluc
FLAG-FZD6-Nluc P427F6.43

**Supplementary Figure 11**. Raw values of Nluc-DVL2 recruitment of SNAP-tagged $FZD_4$, $FZD_5$, $FZD_6$, $FZD_7$ and SMO and their corresponding Pro/Phe$^{6.43}$ mutants. Bystander BRET ratio changes (between Nluc–DVL2 and Venus–KRas) were assessed in $\Delta FZD_{1\text{-}10}$ HEK293 cells in the presence of overexpressed wild type and mutated SNAP–tagged $FZD_4$, $FZD_5$, $FZD_6$ and $FZD_7$. Data are presented as mean ± SEM of 4 independent experiments. These data were normalized to the surface expression of the individual receptor constructs (see Supplementary Fig. 6e) to generate data presented in Fig. 5b as described in the data analysis section. Data were analysed for each receptor wild type/mutant using paired two-tailed t-test. *$P$ < 0.05, **$P$ < 0.01. P = 0.0024 for $FZD_5$, $P$ = 0.0027 for $FZD_6$ and $P$ = 0.0118 for $FZD_7$. Source data are provided as a Source Data file.

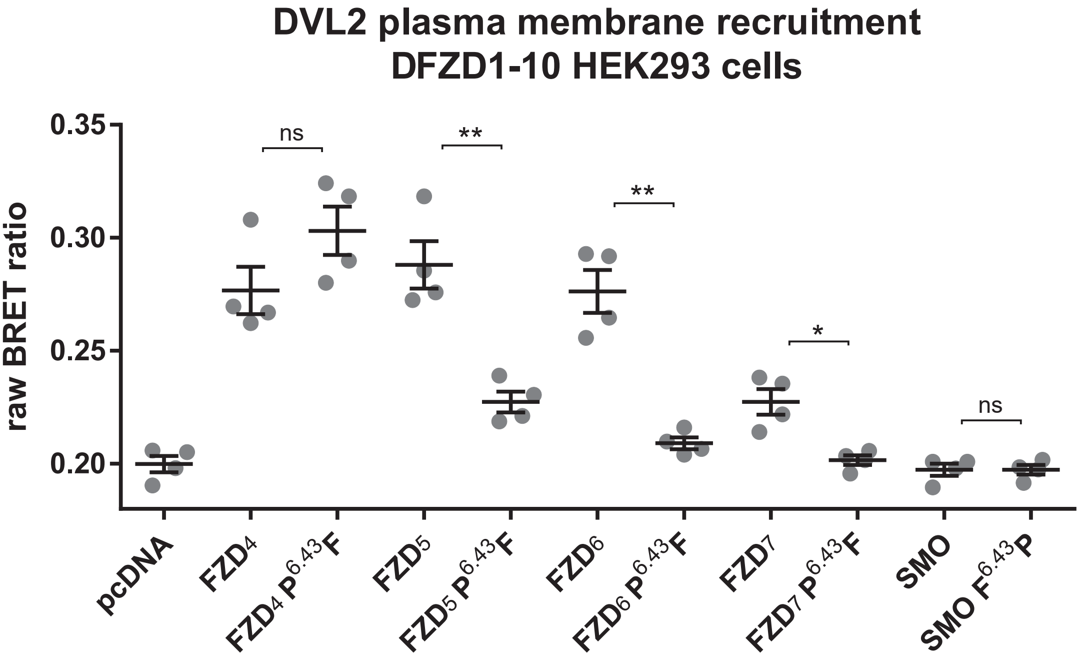

DVL2 plasma membrane recruitment
DFZD1-10 HEK293 cells
raw BRET ratio
0.35
0.30
0.25
0.20
ns
**
**
*
ns
pcDNA
FZD4
FZD4 P6.43F
FZD5
FZD5 P6.43F
FZD6
FZD6 P6.43F
FZD7
FZD7 P6.43F
SMO
SMO F6.43P

**Supplementary Figure 12**. Cholesterol tunnel in SMO and lack of thereof in FZDs. The whole 7TM binding pocket (volume at isovalue 1; represented as purple grid) of $FZD_6$ (left panels) and SMO (right panels). The location of the cholesterol 'gate' is marked with pink arrows (upper panels) and pink circles (lower panels). The receptors are shown as cartoon (white and violet for $FZD_6$ and SMO, respectively) and the amino acid residues forming the 'gate' as sticks. Color code is as follows: red, oxygen; blue, nitrogen; light violet, carbon.

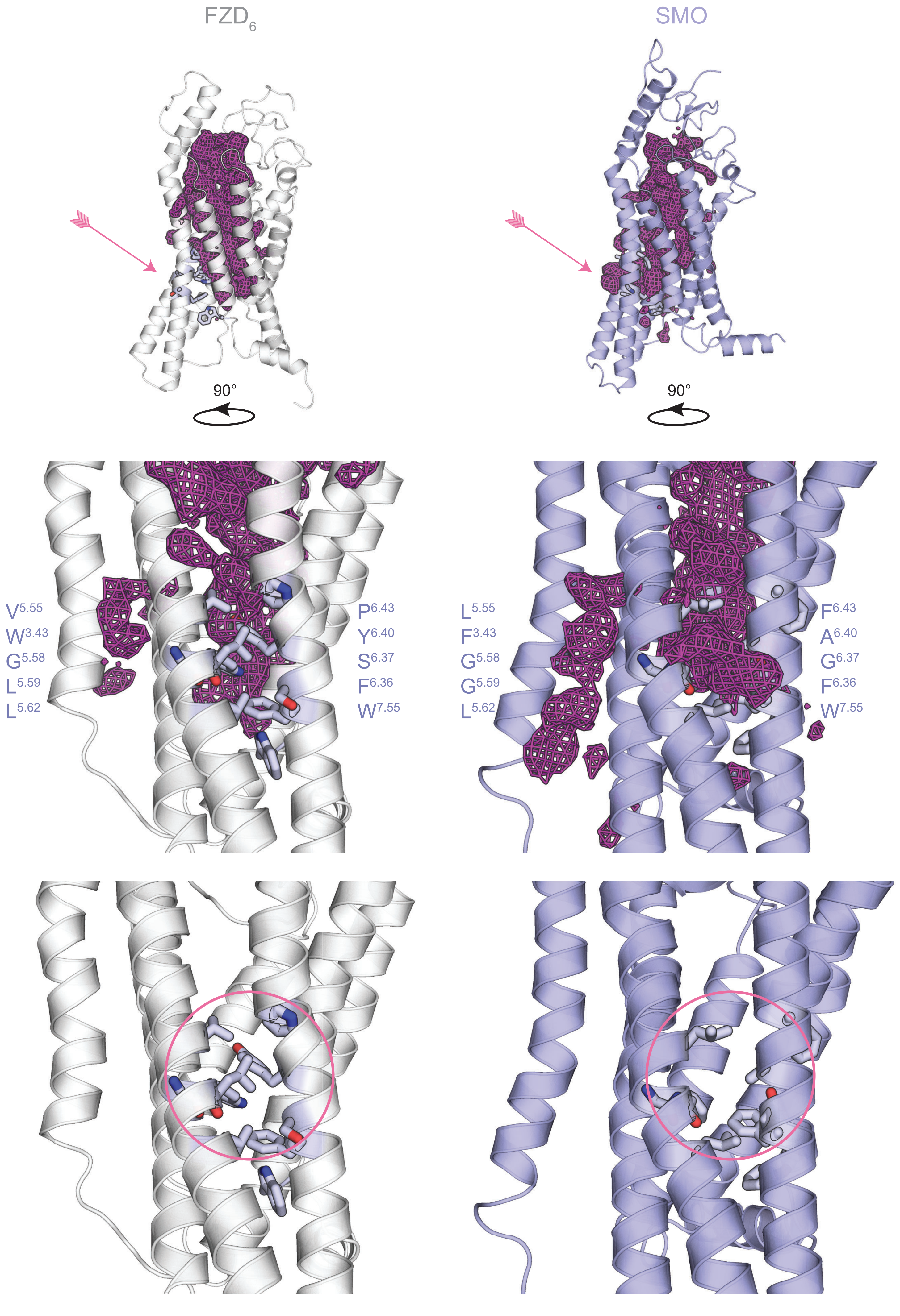

FZD6
SMO
90°
90°
V5.55
W3.43
G5.58
L5.59
L5.62
P6.43
Y6.40
S6.37
F6.36
W7.55
L5.55
F3.43
G5.58
G5.59
L5.62
F6.43
A6.40
G6.37
F6.36
W7.55

**Supplementary Table 1.** List of primers used in the study.

| name | sequence 5' - 3' |
|---|---|
| Hindlll_kozak_ss_HiBiT _BamHI_fwd | AGC TTG CCA CCA TGC GGC TCT GCA TCC CGC AGG TGC TGT TGG CCT TGT TCC TTT CCA TGC TGA CAG GGC CGG GAG AAG GCA GCC GGG TGA GCG GCT GGC GGC TGT TCA AGA AGA TTA GCG |
| Hindlll_kozak_ss_HiBiT _BamHI_rev | GAT CCG CTA ATC TTC TTG AAC AGC CGC CAG CCG CTC ACC CGG CTG CCT TCT CCC GGC CCT GTC AGC ATG GAA AGG AAC AAG GCC AAC AGC ACC TGC GGG ATG CAG AGC CGC ATG GTG GCA |
| FZD4_P447F_fwd | GTA CTG TAC ACA GTT TTT GCA ACG TGT GTG AT |
| FZD4_P447F_rev | ATC ACA CAC GTT GCA AAA ACT GTG TAC AG TAC |
| mFZD5_P460F_fwd | CTG CTC TAC ACG GTG TTT GCC AGC ATC GTG GTG |
| mFZD5_P460F_rev | CAC CAC GAT GCT GGC AAA CAC CGT GTA GAG CAG |
| FZD6_P427F_fwd | GGC TTG TAT CTT GTG TTC TTA GTG ACA CTT CTC |
| FZD6_P427F_rev | GAG AAG TGT CAC TAA GAA CAC AAG ATA CAA GCC |
| FZD7_P481F_fwd | GTG CTC TAC ACA GTG TTC GCC ACC ATC GTC CT |
| FZD7_P481F_rev | AGG ACG ATG GTG GCG AAC ACT GTG TAG AGC AC |
| mSMO_F466P_fwd | TTC CTG GCC TTT GGC CCT GTG CTC ATC ACC TT |
| mSMO_F466P_rev | AAG GTG ATG AGC ACA GGG CCA AAG GCC AGG AA |
| FLAG-SNAP-mSMO-Nluc_VF | CGA ACG CAT TCT GGC GTA ATC TAG AGG GCC CGT T |
| FLAG-SNAP-mSMO-Nluc_VR | GGT GGC GAC CGG TTG GAA GTC CGA GTC TGC A |
| FLAG-SNAP-mSMO-Nluc_IF | GCA GAC TCG GAC TTC CAA CCG GTC GCC ACC |
| FLAG-SNAP-mSMO-Nluc_IR | AAC GGG CCC TCT AGA TTA CGC CAG AAT GCG TTC G |

## References


1 Wright, S. C. *et al*. A conserved molecular switch in Class F receptors regulates receptor activation and pathway selection. *Nat Commun* **10**, 667, doi:10.1038/s41467-019-08630-2 (2019).

2 Wu, D. The puckering free-energy surface of proline. *AIP Advances* **3**, 032141, doi:10.1063/1.4799082 (2013).

3 Milner-White, E. J., Bell, L. H. & Maccallum, P. H. Pyrrolidine ring puckering in cis and trans-proline residues in proteins and polypeptides: Different puckers are favoured in certain situations. *Journal of Molecular Biology* **228**, 725-734, doi:10.1016/0022-2836(92)90859-I (1992).
