# Supplementary material for: Residue 6.43 defines receptor function in class F GPCRs
Source: Nat Commun. 2021 Jun 24;12:3919. doi: 10.1038/s41467-021-24004-z (PMC8225760; doi:10.1038/s41467-021-24004-z)
Supplement: Supplementary file 3 — Description of Additional Supplementary Files [file 41467_2021_24004_MOESM3_ESM.pdf]

Description of Additional Supplementary Files

File Name: Supplementary Data 1
Description: Sequence alignment of class F GPCRs. The sequences were fetched from the Uniprot/SwissProt database, aligned with ClustalX2 and visualized in UGENE. The TM helices and loops are annotated with horisontal boxes and arrows, respectively. The amino acid residues discussed in this study are marked with pink arrows and labelled with Ballesteros-Weinstein numbering.

File Name: Supplementary Data 2
Description: MD simulation snapshots for FZD6 wild type simulation replica 1 (frames output every 50th ns of the simulation). The first frame corresponds to t=0.

File Name: Supplementary Data 3
Description: MD simulation snapshots for FZD_6 wild type simulation replica 2 (frames output every 50th ns of the simulation). The first frame corresponds to t=0.

File Name: Supplementary Data 4
Description: MD simulation snapshots for FZD_6 wild type simulation replica 3 (frames output every 50th ns of the simulation). The first frame corresponds to t=0.

File Name: Supplementary Data 5
Description: MD simulation snapshots for FZD6 wild type simulation replica 4 (frames output every 50th ns of the simulation). The first frame corresponds to t=0.

File Name: Supplementary Data 6
Description: MD simulation snapshots for FZD_6 $P^{6.43}F$ simulation replica 1 (frames output every 50th ns of the simulation). The first frame corresponds to t=0.

File Name: Supplementary Data 7
Description: MD simulation snapshots for FZD6 $P^{6.43}F$ simulation replica 2 (frames output every 50th ns of the simulation). The first frame corresponds to t=0.

File Name: Supplementary Data 8
Description: MD simulation snapshots for FZD6 $P^{6.43}F$ simulation replica 3 (frames output every 50th ns of the simulation). The first frame corresponds to t=0.

File Name: Supplementary Data 9
Description: MD simulation snapshots for FZD6 $P^{6.43}F$ simulation replica 4 (frames output every 50th ns of the simulation). The first frame corresponds to t=0.

File Name: Supplementary Data 10
Description: MD simulation snapshots for SMO wild type simulation replica 1 (frames output every 50th ns of the simulation). The first frame corresponds to t=0.

File Name: Supplementary Data 11
Description: MD simulation snapshots for SMO wild type simulation replica 2 (frames output every 50th ns of the simulation). The first frame corresponds to t=0.

File Name: Supplementary Data 12
Description: MD simulation snapshots for SMO wild type simulation replica 3 (frames output every 50th ns of the simulation). The first frame corresponds to t=0.

File Name: Supplementary Data 13
Description: MD simulation snapshots for SMO wild type simulation replica 4 (frames output every 50th ns of the simulation). The first frame corresponds to t=0.

File Name: Supplementary Data 14
Description: MD simulation snapshots for SMO $F^{6.43}P$ simulation replica 1 (frames output every 50th ns of the simulation). The first frame corresponds to t=0.

File Name: Supplementary Data 15
Description: MD simulation snapshots for SMO $F^{6.43}P$ simulation replica 2 (frames output every 50th ns of the simulation). The first frame corresponds to t=0.

File Name: Supplementary Data 16
Description: MD simulation snapshots for SMO $F^{6.43}P$ simulation replica 3 (frames output every 50th ns of the simulation). The first frame corresponds to t=0.

File Name: Supplementary Data 17
Description: MD simulation snapshots for SMO $F^{6.43}P$ simulation replica 4 (frames output every 50th ns of the simulation). The first frame corresponds to t=0.

File Name: Supplementary Data 18
Description: MD simulation snapshots for FZD_6 wild type – miniGi complex simulation replica 1 (frames output every 50th ns of the simulation). The first frame corresponds to t=0.

File Name: Supplementary Data 19
Description: MD simulation snapshots for FZD_6 wild type – miniGi complex simulation replica 2 (frames output every 50th ns of the simulation). The first frame corresponds to t=0.

File Name: Supplementary Data 20
Description: MD simulation snapshots for FZD_6 wild type – miniGi complex simulation replica 3 (frames output every 50th ns of the simulation). The first frame corresponds to t=0.

File Name: Supplementary Data 21
Description: An example script for measuring the distances between the mass centres of the residues of the aromatic network throughout the simulation trajectories using CPPTRAJ package (AmberTools 18).
